# Comparative Adsorption of Mono‑, Di‑, and Tricarboxylic Acids on Magnetically Modified Graphene Oxide: Influence of Molecular Structure on Adsorption Behavior

**DOI:** 10.1021/acsomega.6c05759

**Published:** 2026-09-10

**Authors:** Ebubekir Ekinci, Hasan Uslu, Şahika Sena Bayazit, Mehmet Yetişen

**Affiliations:** † Engineering Faculty, Food Engineering Department, Nigde Omer Halisdemir University, Niğde 51240, Türkiye; ‡ Institute of Nanotechnology and Biotechnology, İstanbul University-Cerrahpasa, İstanbul 34500, Türkiye

## Abstract

This study investigates the efficient adsorption and recovery of biotechnologically significant organic acids (lactic, succinic, and citric acids) using a magnetically modified graphene oxide (MGO) composite. The MGO was synthesized via chemical coprecipitation, and its structural, functional, and textural properties were comprehensively characterized using X-ray diffraction (XRD), Fourier transform infrared spectroscopy (FTIR), and Brunauer–Emmett–Teller (BET) analysis. Nitrogen adsorption–desorption measurements revealed a mesoporous structure with a specific surface area of 37.94 m^2^/g, a total pore volume of 0.078 cm^3^/g, and an average pore radius of 4.12 nm, which facilitate effective molecular diffusion into the internal pore framework. Batch adsorption experiments were conducted at 25 °C using an adsorbent dosage of 10 mg in a 20 mL solution volume across a concentration range of 10–100 g/L to evaluate equilibrium performance in comparison with a magnetically modified activated carbon (AC) reference. The equilibrium times were established as 50–80 min depending on the specific organic acid. Adsorption kinetics were accurately described by the pseudo-second-order (PSO) model (*R* ^2^ > 0.99), confirming a chemisorption-controlled mechanism for all investigated acids. To ensure physical consistency and rigorous mass balance reconciliation, adsorption capacities were evaluated using mass-normalized (mg/g) units. The equilibrium data were analyzed using Langmuir, Freundlich, and Temkin isotherm models. While the results showed excellent agreement with the Langmuir model, the calculated dimensionless separation factor (*R*_L_) values (ranging from 2.3 × 10^–6^ to 2.8 × 10^–3^) further confirmed that the adsorption process is highly favorable for all investigated acids across the concentration range. Lactic acid exhibited the highest maximum adsorption capacity (*q*_max_ = 334.45 mg/g), which is attributed to its smaller molecular size and the specific interaction of its hydroxyl (−OH) group, enabling enhanced hydrogen bonding and electrostatic interactions with the oxygen-rich surface sites of the MGO. Furthermore, regeneration studies demonstrated that the MGO composite maintains a high capacity retention rate of 91.4% after five consecutive cycles, confirming its excellent structural stability and long-term industrial feasibility. The results demonstrate that the MGO composite provides superior adsorption performance compared to AC and allows for rapid magnetic separation from aqueous solutions. This study underscores the potential of functionalized graphene-based materials for the sustainable separation and purification of platform chemicals in the biotechnology industry.

## Graphical abstract

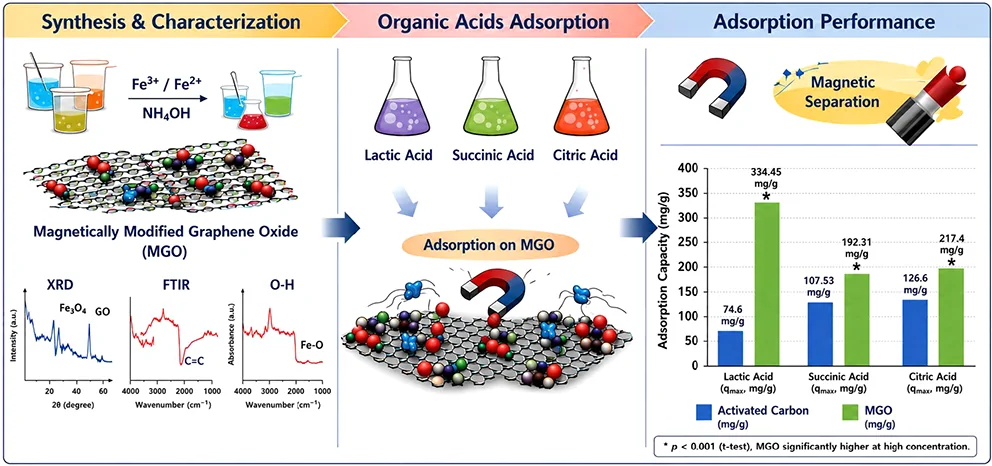

## 1 Introduction

Carbon exists in several allotropes, including graphite-, diamond-, and graphene-based nanostructures, each exhibiting distinct structural and physicochemical properties. Graphene, a single atomic layer of sp^2^-hybridized carbon atoms arranged in a hexagonal lattice, has attracted extensive scientific attention since its isolation because of its exceptional electrical conductivity, mechanical strength, and high specific surface area.^1–3^ Among graphene-derived materials, graphene oxide (GO) has emerged as a particularly promising functional material owing to the presence of abundant oxygen-containing functional groups such as hydroxyl, epoxy, carbonyl, and carboxyl moieties on its basal plane and edges. These functionalities enhance the hydrophilicity and provide active sites suitable for adsorption and surface modification.^4^

Adsorption processes play a crucial role in the separation and purification of valuable compounds in chemical, environmental, and biotechnological industries due to their simplicity, cost-effectiveness, and operational flexibility.^5,6^ In particular, the recovery and purification of organic acids are of significant industrial importance, as these compounds are widely used as food additives, preservatives, and intermediates in biotechnological production systems.^7,8^ Various separation and recovery techniques have been developed for organic acids, including reactive extraction, membrane separation, ion exchange, and adsorption. Reactive extraction has attracted considerable attention because of its high extraction efficiency and suitability for dilute fermentation broths; however, it generally requires organic solvents or extractants, followed by additional stripping and solvent regeneration steps, which increase the process complexity and operational cost.^9–11^ Membrane-based processes provide high separation efficiency and continuous operation; however, challenges such as fouling remain. Recent innovations, such as the anhydrous interfacial polymerization (AIP) of polyamide membranes, have demonstrated the potential for precise ionic sieving with sub-1 Å accuracy, offering a high-precision alternative for aqueous separation.^12^ Nevertheless, concentration polarization and relatively high capital costs remain important limitations for large-scale applications.^13–15^ Ion-exchange resins exhibit high selectivity and regeneration capability for charged compounds; nevertheless, their performance strongly depends on solution chemistry, and regeneration often requires significant quantities of chemicals, which may increase operating costs and generate secondary waste streams.^16–18^ In contrast, adsorption is considered a simple, flexible, and economically attractive separation technique owing to its operational simplicity, relatively low energy requirement, and ease of regeneration, making it particularly suitable for the recovery and purification of valuable compounds from aqueous systems.^19,20^ Consequently, the development of high-performance adsorbents with enhanced adsorption capacity, selectivity, and facile recovery has become an important research focus. Conventional adsorbents such as activated carbon (AC) are widely employed;^21^ however, challenges including limited selectivity, regeneration difficulties, and separation efficiency motivate the development of advanced adsorbent materials.

GO has attracted increasing interest as an adsorbent due to its large specific surface area and versatile surface chemistry, enabling strong interactions with various organic molecules.^22–24^ Advanced graphene-based materials obtained through chemical modification or doping strategies have shown improved adsorption efficiency due to increased structural defects, active sites, and surface functionality, emphasizing the role of tailored surface chemistry in adsorption applications.^25^ Nevertheless, the practical application of GO is often limited by difficulties in post-treatment separation from aqueous systems. To address this limitation, magnetic modification using Fe_3_O_4_ nanoparticles to produce magnetically modified graphene oxide (MGO) has been widely explored.^26,27^ Graphene oxide-based systems have demonstrated strong adsorption behavior and structural stabilization capabilities through π–π interactions and surface functional groups, which may enhance protection mechanisms in encapsulation or matrix-based delivery systems.^28^ Compared with reactive extraction, membrane separation, and ion-exchange processes, adsorption using MGO offers several practical advantages, including a large specific surface area, abundant oxygen-containing functional groups, rapid adsorption kinetics, and simple magnetic separation without the need for additional filtration or centrifugation steps. Furthermore, the magnetic nature of MGO facilitates adsorbent recovery and reuse, making it an attractive and sustainable alternative for the selective recovery of organic acids from aqueous media.^19,20^

Biotechnologically relevant organic acids with different numbers of carboxyl functional groups may exhibit distinct adsorption behaviors depending on the molecular structure and surface interactions.^29^ Despite the growing body of literature, a systematic comparison of how functional group density and molecular complexity (mono-, di-, and tricarboxylic) influence the adsorption mechanism on a single MGO platform remains limited. The novelty of this study lies in the detailed investigation of the comparative adsorption behavior of three structurally distinct organic acids on a verified mesoporous MGO framework. By reconciling mass balance through standardized titration methods and evaluating multiple isotherm models (Langmuir, Freundlich, and Temkin), this work provides new mechanistic insights into the role of specific interactions such as the hydroxyl group influence on lactic acid and the efficacy of graphene-based composites. Ultimately, this systematic approach contributes to the rational design of adsorbents for the sustainable and selective recovery of platform chemicals in large-scale biotechnological processes.

## 2 Materials and Methods

### 2.1 Materials

GO and AC were utilized as the primary adsorbent materials. Analytical-grade ferric chloride (FeCl_3_), ferrous chloride tetrahydrate (FeCl_2_·4H_2_O), ammonia solution (25%), hydrochloric acid (HCl), acetic acid, methanol, and diethylamine (DEA) were used for the synthesis and modification processes. For adsorption studies, lactic acid (monocarboxylic), succinic acid (dicarboxylic), and citric acid (tricarboxylic) were obtained at analytical purity. Sodium hydroxide (NaOH) solutions used for titration were standardized with potassium hydrogen phthalate, and phenolphthalein was employed as the indicator. All solutions were prepared using deionized water (resistivity > 18.2 MΩ cm).

### 2.2 Synthesis of MGO

The MGO composite was synthesized via a modified chemical coprecipitation route. Briefly, 0.5 g of GO was dispersed in 60 mL of deionized water and subjected to ultrasonication (25 °C, 20 min) to ensure a homogeneous suspension. Subsequently, iron precursors (0.083 g of FeCl_3_ and 0.042 g FeCl_2_ 4H_2_O) were added. The initial pH was adjusted to 2.13 using 0.1 mL of concentrated HCl. Magnetite (Fe_3_O_4_) precipitation was induced by the dropwise addition of ammonia solution until a pH of 10.11 was reached. The mixture was allowed to equilibrate for 1 h, and the resulting magnetic precipitate was washed repeatedly with deionized water until a neutral pH (7.0) was achieved to remove residual ions.

For surface functionalization, 1.1322 g of the wet precipitate was treated with a mixture of acetic acid (22.6 mL), methanol (22.6 mL), and diethylamine (9.56 mL). The suspension was dried at 100 °C, yielding approximately 0.2008 g of the final MGO product. Diethylamine was introduced to promote nanoparticle anchoring and provide surface stabilization through coordination interactions. To minimize potential analytical interference from residual amines during subsequent acid titrations, the composite underwent extensive washing cycles until the supernatant reached a neutral pH. The same protocol was applied to AC to prepare the AC-based magnetic reference.

### 2.3 Characterization Techniques

The crystalline structure and functional groups of MGO were identified using X-ray diffraction (XRD; PANalytical-EMPYREAN) and Fourier transform infrared spectroscopy (FTIR; Bruker VERTEX 70, Germany), respectively. Furthermore, to meet the standards for porous materials, the textural properties were characterized by adsorption–desorption measurements using a Quantachrome NovaTouch 4LX analyzer. The specific surface area was determined via the multipoint Brunauer–Emmett–Teller (BET) method, while the total pore volume and average pore radius were calculated based on the adsorption branch of the isotherm.

### 2.4 Adsorption Experiments

All adsorption experiments for lactic, succinic, and citric acids were conducted using a standardized protocol to ensure high comparability between the systems. The experiments were performed at 25 °C with an agitation speed of 120 rpm, utilizing 10 mg of adsorbent in a 20 mL solution volume. For the sake of physical consistency and precise mass balance, all initial acid concentrations were converted from % (w/v) to standard mass units (g/L or mg/L) for all calculations. Accordingly, the investigated concentrations of 1, 2.5, 5, 7.5, and 10% (w/v) correspond to 10, 25, 50, 75, and 100 g/L. The pH of each solution was monitored at the start and at equilibrium to evaluate the effect of acid speciation on adsorption performance. The experiments were conducted at the natural pH of the organic acid solutions (ranging from approximately 2.0 to 3.5), which is generally near or below the p*K*_a_ values of the investigated acids (lactic acid p*K*_a_ = 3.86, succinic acid p*K*_a1_ = 4.21, and citric acid p*K*_a1_ = 3.13). At this specific pH range, the organic acid molecules exist predominantly in their neutral (undissociated) forms. This state is critical as it minimizes electrostatic repulsion between the acids and the MGO surface, thereby promoting recovery through hydrogen bonding and π–π interactions.^30,31^ This neutralization of the molecular charge effectively eliminates the electrostatic repulsion barrier, allowing for an unhindered approach of the organic acid molecules to the MGO surface and ensuring that hydrogen bonding with the oxygen-rich moieties becomes the primary and most efficient interaction mechanism. The relationship between the solution pH and dissociation state of the organic acids was analyzed to provide a mechanistic basis for the surface interactions.

#### 2.4.1 Determination of Equilibrium Time and Analytical Procedures

Adsorption studies were performed by using succinic, citric, and lactic acids as model adsorbates at a 5% (w/v) initial concentration (50 g/L). The mixtures were incubated in a shaking incubator at 120 rpm. To ensure accurate titration and eliminate interference from suspended MGO particles or potential iron leaching, the adsorbents were separated by using an external permanent magnet followed by centrifugation.

The acid concentration was determined via titration with standardized 0.1 N NaOH. For each analysis, 1 mL aliquots were diluted with 9 mL of deionized water to ensure that the titration volume remained within an ideal range. Blank titrations were performed with MGO in deionized water to account for potential basicity arising from residual diethylamine or iron species, ensuring that the recorded adsorption was not an artifact of neutralization. All measurements were performed in triplicate to report the mean values and standard deviations. Equilibrium times were established as 50 min for succinic acid, 60 min for citric acid, and 80 min for lactic acid.

#### 2.4.2 Adsorption Isotherm Studies

Isotherm studies were conducted across the concentration range of 10–100 g/L based on the predetermined equilibrium times. To reconcile the mass balance and resolve previous numerical inconsistencies, the adsorption capacity at equilibrium (*Q*_e_) was calculated using the following equation

1 $$Q_{e}=\frac{\left(C_{0}-C_{e}\right)\times V}{m}$$

where *Q*_e_ is the adsorption capacity (mg/g), *C*_0_ is the initial concentration (mg/L), *C*_e_ is the equilibrium concentration (mg/L), *V* is the solution volume (L), and *m* is the adsorbent mass (g). For illustrative purposes, the calculation of *Q*_e_ is performed as follows: an initial concentration of 1% (w/v) corresponds to 10,000 mg/L. Using a solution volume (*V*) of 0.02 L and an adsorbent mass (*m*) of 0.01 g, if the measured equilibrium concentration (*C*_e_) is 9832.78 mg/L, the resulting adsorption capacity (*Q*_e_) is calculated as follows: *Q*_e_ = (10,000–9832.78) × 0.02/0.01 = 334.44 mg/g. This example is provided to ensure clarity in unit conversion and mass balance reconciliation, corresponding to the experimental range observed for lactic acid in this study.

#### 2.4.3 Regeneration and Reusability Studies

To evaluate the sustainable application potential of the MGO composite, cyclic adsorption–desorption experiments were conducted using lactic acid as the model adsorbate. After each adsorption cycle, the MGO was separated from the solution using an external magnetic field. The spent adsorbent was then treated with 0.1 M NaOH as a desorption agent, followed by washing with deionized water and ethanol until a neutral pH was achieved. The regenerated MGO was dried at 60 °C and reused in five consecutive adsorption cycles. The capacity retention rate was calculated by comparing the *q*_e_ of each cycle to the initial adsorption capacity.

### 2.5 Adsorption Isotherm Modeling

To provide a comprehensive understanding of the equilibrium interaction between the organic acids and the adsorbents (MGO and AC), we analyzed the adsorption data using three fundamental isotherm models: Langmuir, Freundlich, and Temkin. In response to technical requirements for physical consistency and mass balance reconciliation, all experimental data originally recorded in % (w/v) were converted to standard units of equilibrium concentration (*C*_e_, mg/L) and adsorption capacity (*Q*_e_, mg/g) prior to model fitting.

#### 2.5.1 Langmuir Isotherm

The Langmuir model assumes that adsorption occurs on a homogeneous surface via monolayer coverage, where all adsorption sites are identical, and no interactions between adsorbed molecules occur. The linear form of the Langmuir equation is expressed as

2 $$\frac{C_{e}}{Q_{e}}=\frac{1}{K_{L}q_{\max}}+\frac{C_{e}}{q_{\max}}$$

where *Q*_e_ (mg/g) is the adsorption capacity at equilibrium, *C*_e_ (mg/L) is the equilibrium concentration, *q*_max_ (mg/g) is the theoretical maximum monolayer adsorption capacity, and *K*_L_ (L/mg) is the Langmuir constant related to the affinity of the binding sites. To assess the feasibility of the adsorption process, the dimensionless separation factor (*R*_L_) was calculated using the following equation

3 $$R_{L}=\frac{1}{1+K_{L}C_{0}}$$

where *C*_0_ is the highest initial acid concentration (mg L). The value of *R*_L_ indicates whether the adsorption is unfavorable (*R*_L_ > 1), linear (*R*_L_ = 1), favorable (0 < *R*_L_ < 1), or irreversible (*R*_L_ = 0).

#### 2.5.2 Freundlich Isotherm

The Freundlich isotherm is an empirical model used to describe multilayer adsorption on heterogeneous surfaces where the adsorption heat is nonuniformly distributed. Its linear form is given by

4 $${\ln Q}_{e}={\ln K}_{F}+\frac{1}{n}{\ln C}_{e}$$

where *K*_F_ [(mg/g)·(L/mg)^1/*n*^] represents the adsorption capacity and 1/*n* is the heterogeneity factor. A value of 1/*n* between 0 and 1 indicates a favorable adsorption process.

#### 2.5.3 Temkin Isotherm

The Temkin model accounts for the effects of indirect adsorbent–adsorbate interactions, assuming that the heat of adsorption decreases linearly rather than logarithmically with surface coverage. The linear equation is

5 $$Q_{e}=B\ln A+B\ln C_{e}$$

where *A* (L/g) is the equilibrium binding constant, and *B* is a constant related to the heat of adsorption.

#### 2.5.4 Adsorption Kinetics Models

To investigate the adsorption mechanism and identify the rate-controlling steps, the experimental data for the recovery of lactic, succinic, and citric acids were analyzed using the pseudo-second-order (PSO) and intraparticle diffusion (IPD) models. The linear form of the pseudo-second-order (PSO) kinetic model is expressed as follows

6 $$\frac{t}{q_{t}}=\frac{1}{k_{2}\times q_{e}^{2}}+\left(\frac{1}{q_{e}}\right)t$$

where *q*_e_ and *q*_*t*_ (mg/g) are the amounts of organic acid adsorbed at equilibrium and at any time *t* (min), respectively, and *k*_2_ (g/mg·min) represents the second-order adsorption rate constant. The values of *q*_e,cal_ and *k*_2_ are determined from the slope and intercept of the linear plot of *t*/*q*_*t*_ versus *t*.

The intraparticle diffusion (IPD) model was further applied to elucidate the diffusion mechanism, according to the following equation

7 $$q_{t}=k_{id}\times t^{0.5}+C$$

where *k*_id_ (mg/g·min) is the intraparticle diffusion rate constant, and the intercept *C* (mg/g) is a constant related to the thickness of the boundary layer. A multilinear plot of *q*_*t*_ versus *t* ^0.5^ indicates that the adsorption process involves multiple stages, including external surface adsorption and internal pore diffusion.

### 2.6 Statistical Analysis

To evaluate the statistical significance of the comparative adsorption performance between MGO and magnetic AC, a two-sample *t*-test was performed on the experimental data sets. The statistical analysis was conducted using Minitab 21.4.1 software (USA). A *p*-value of less than 0.05 (*p* < 0.05) was considered to indicate statistical significance in the comparative evaluation of adsorption capacities (*Q*_e_) and equilibrium concentrations (*C*_e_).

## 3 Results and Discussion

### 3.1 FTIR Analysis

FTIR spectroscopy was used to identify the functional groups present in the synthesized materials. The FTIR spectrum is shown in Figure 1.

**Figure 1:**
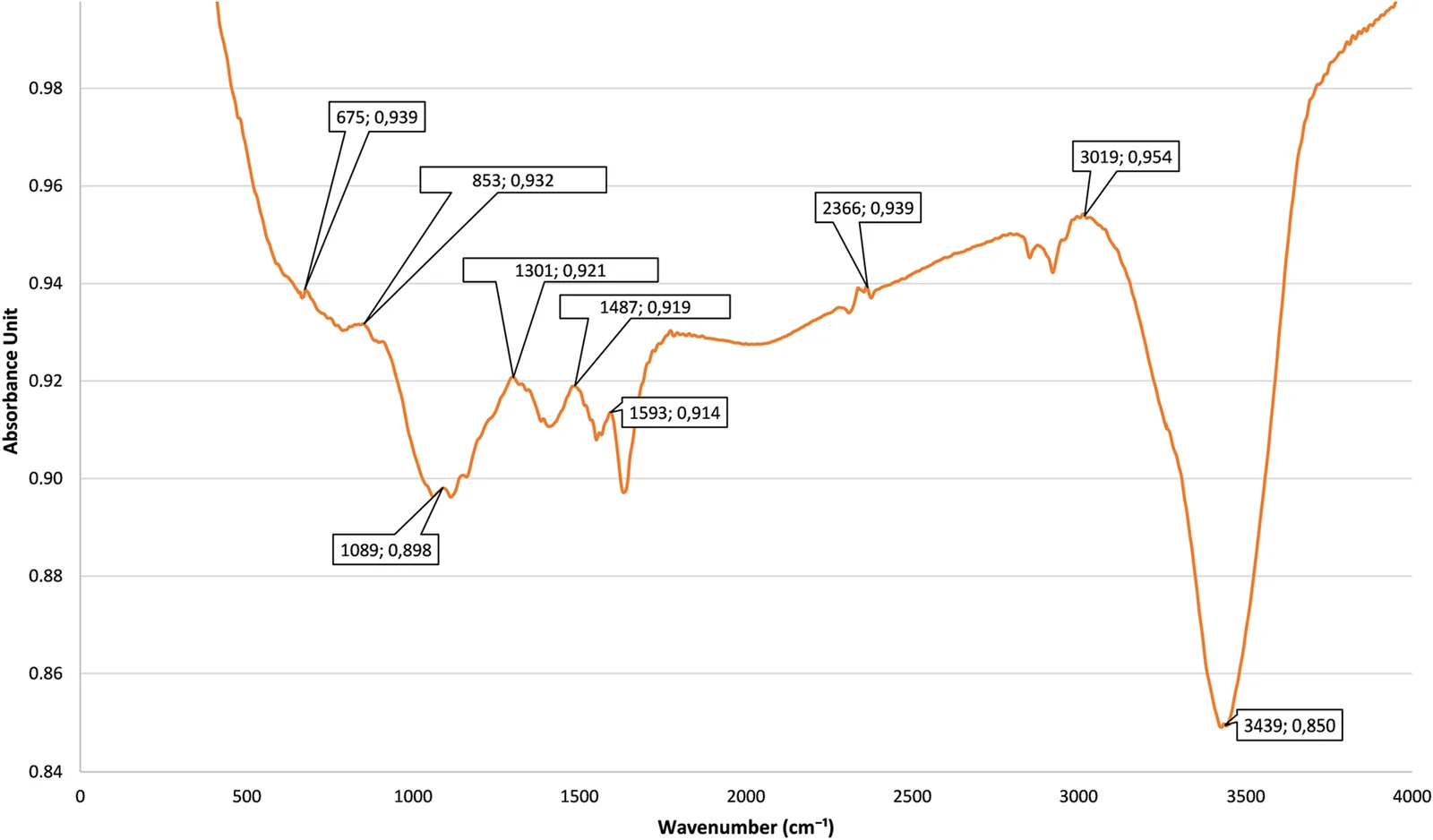
FTIR spectrum of MGO.

The FTIR spectrum of the MGO composite exhibited several characteristic absorption bands consistent with oxygen-functionalized graphene materials and iron oxide incorporation. A broad absorption band centered at approximately 3439 cm^–1^ was assigned to O–H stretching vibrations, corresponding to hydroxyl groups and adsorbed moisture commonly observed in graphene oxide-based materials. The band at 3019 cm^–1^ may be associated with C–H stretching vibrations. Bands observed at 1593 and 1487 cm^–1^ are attributed to skeletal vibrations of the graphitic carbon framework and aromatic C=C stretching. The band near 1301 cm^–1^ can be assigned to C–O stretching vibrations of epoxy and alkoxy functionalities, while the feature around 1089 cm^–1^ is consistent with C–O stretching of oxygen-containing groups. A weak low-wavenumber feature was observed in the region below 700 cm^–1^, which may indicate Fe–O vibrations associated with iron oxide incorporation; however, this region was not sufficiently resolved for definitive assignment. The persistence of O–H- and C–O-related bands indicates that oxygen-containing functional groups were retained after magnetic modification, suggesting preservation of the graphene oxide structure. In combination with XRD results, these observations support successful incorporation of iron oxide species into the graphene oxide matrix. These results are consistent with graphene oxide-based materials reported in the literature.^32^ Compared with pristine graphene oxide reported in previous studies, the observed spectral changes, including the slight broadening of the O–H stretching band and the presence of Fe–O vibrations, are consistent with the successful deposition of Fe_3_O_4_ nanoparticles onto graphene oxide sheets.^30,33^ Similar FTIR features have been reported for magnetic graphene oxide nanocomposites, where interactions between iron oxide nanoparticles and oxygen-containing functional groups contributed to the formation of stable hybrid structures.^34,35^ Therefore, the present results indicate successful surface modification rather than simple physical mixing.

Similar FTIR characteristics have been reported for GO/Fe_3_O_4_ nanocomposites synthesized by coprecipitation, where the coexistence of O–H, C–O, graphitic C=C, and Fe–O bands confirmed the successful deposition of Fe_3_O_4_ nanoparticles onto graphene oxide sheets while preserving the oxygen-containing functional groups responsible for surface reactivity.^30^ Likewise, piperazine-functionalized magnetic graphene oxide exhibited comparable spectral features, with the retention of oxygen-containing functional groups after magnetic modification indicating that the graphene oxide framework remained intact during synthesis.^33^

Comparable observations have also been reported for other magnetic graphene oxide-based nanocomposites prepared for biomedical applications. In GO/Fe_3_O_4_-modified clay nanocomposites, FTIR analysis confirmed that the characteristic oxygen-containing functional groups of graphene oxide were preserved after incorporation of Fe_3_O_4_ nanoparticles, indicating strong interfacial interactions without disruption of the graphene structure.^34^ Similarly, MGO-containing hydrogel scaffolds exhibited broad O–H absorption bands associated with extensive hydrogen bonding between graphene oxide and the surrounding polymeric matrix, demonstrating that oxygen-containing functional groups remain available for intermolecular interactions after magnetic modification.^36^

The preservation of oxygenated functional groups after incorporation of magnetic nanoparticles has also been observed in other graphene-based magnetic nanocomposites, including ZnFe_2_O_4_-decorated reduced graphene oxide and multifunctional GO@Fe_3_O_4_ hybrid nanostructures, where FTIR spectra confirmed successful hybrid formation while maintaining the functional groups required for further surface interactions and applications.^35,37^ The close agreement between the present FTIR results and previous reports therefore provides additional evidence for the successful synthesis of the MGO composite and the effective interaction between iron oxide nanoparticles and graphene oxide sheets rather than simple physical blending.

Compared with the magnetic activated carbon reference material, the MGO composite retained the characteristic oxygen-containing functional groups of graphene oxide together with Fe–O vibrations arising from magnetite incorporation. Although both magnetic materials exhibit Fe–O-related absorption associated with Fe_3_O_4_, the stronger O–H and C–O bands observed for MGO reflect the oxygen-rich surface chemistry of graphene oxide, which provides abundant anchoring sites for iron oxide nanoparticles. This distinction suggests that the interaction between Fe_3_O_4_ and graphene oxide is stronger than that expected for activated carbon-based supports and contributes to the enhanced structural integration of the composite.^30,33–37^

### 3.2 XRD Analysis

XRD patterns of the graphene oxide-based sample and AC-based sample are presented in Figures 2 and 3, respectively. Figure 2 corresponds to the MGO composite. Figure 3 represents the magnetically modified activated carbon (AC) reference sample.

**Figure 2:**
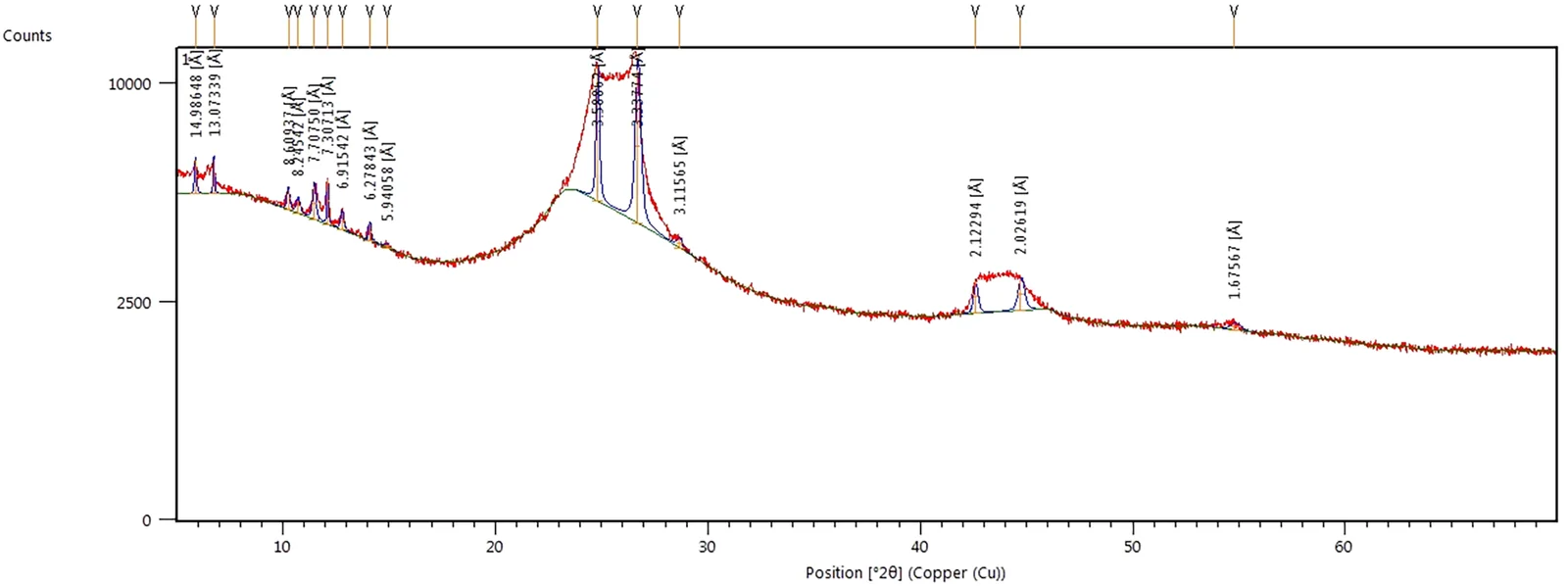
XRD results of MGO.

**Figure 3:**
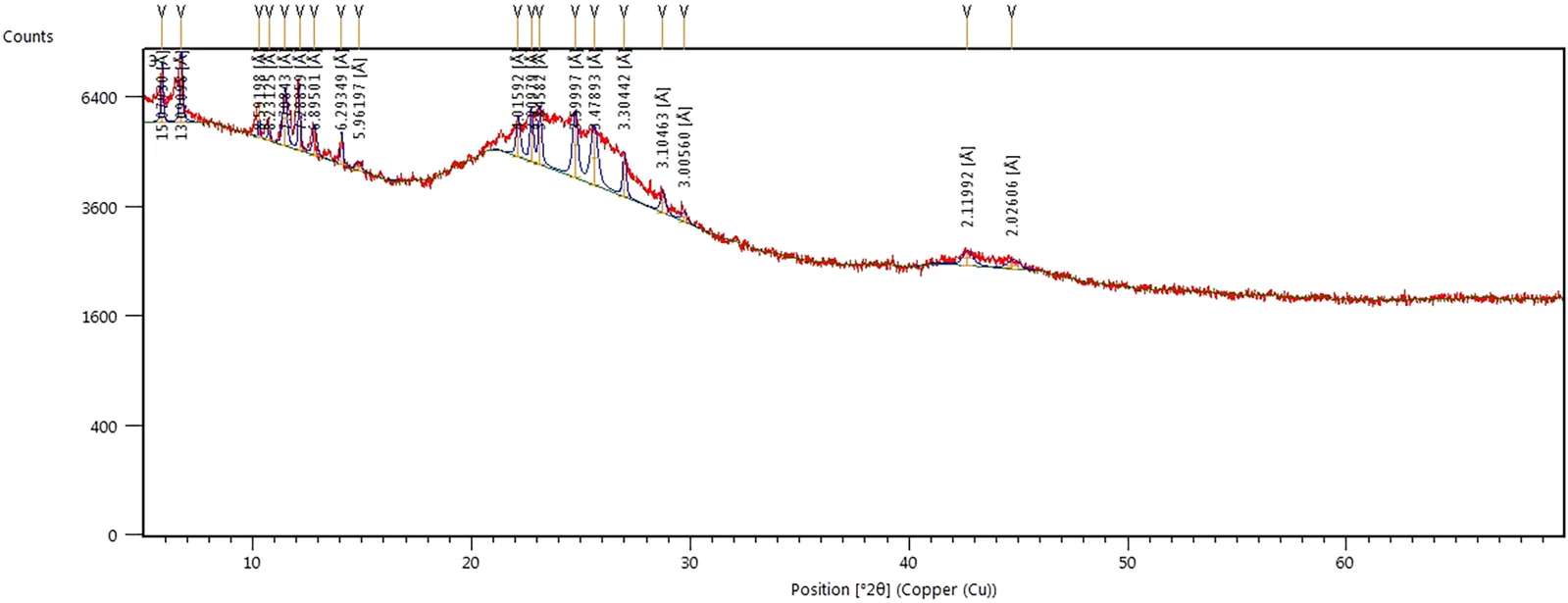
XRD results of magnetically modified AC.

The graphene oxide-based sample exhibits diffraction peaks at approximately 2θ = 25, 27, 42, and 44°, while the AC-based sample shows peaks at 22, 23, 25, 26, 27, 43, and 44°. The similarity between the diffraction patterns in Figures 2 and 3 confirms the presence of graphene within the modified material. These results indicate the successful incorporation of iron species onto the graphene oxide structure. The magnetic behavior of the synthesized composite was qualitatively confirmed through visual magnetic response tests. The material exhibited rapid attraction toward an external permanent magnet, allowing efficient separation from aqueous solution within seconds. This observation supports the successful incorporation of magnetite nanoparticles and confirms the feasibility of magnetic recovery. Beyond structural characterization, the functional integrity of the MGO composite was successfully demonstrated by its rapid magnetic response, achieving complete phase separation from the aqueous solution in less than 30 s under an external magnetic field. This exceptional separation efficiency serves as a robust functional validation of the successful anchoring of magnetite nanoparticles on the GO sheets, confirming that the magnetic phase is effectively integrated to enable practical recovery. The crystalline integrity of the magnetic phase was rigorously validated through the identification of five distinct diffraction peaks at 2θ ≈ 30.1, 35.5, 43.1, 53.5, and 57°, which are precisely indexed to the (220), (311), (400), (422), and (511) crystal planes of Fe_3_O_4_, respectively. The coexistence of these high-intensity magnetite reflections alongside the characteristic graphitic peaks confirms the successful in situ growth of crystalline iron oxide nanoparticles on the graphene oxide matrix, providing a definitive structural fingerprint of the composite formation even in the absence of direct microscopic imaging. Minor peak broadening may indicate reduced crystallite size or interaction between iron oxide nanoparticles and graphene oxide layers.

Similar diffraction patterns have been widely reported for magnetic graphene oxide nanocomposites prepared by coprecipitation methods. In particular, the characteristic reflections of magnetite assigned to the (220), (311), (400), (422), and (511) crystal planes together with the graphene oxide-related diffraction peaks have been considered clear evidence of successful Fe_3_O_4_ deposition onto graphene oxide sheets without altering the crystalline structure of magnetite.^30^ Comparable XRD results were also reported for piperazine-functionalized magnetic graphene oxide, where the coexistence of graphene oxide and Fe_3_O_4_ diffraction peaks confirmed successful magnetic functionalization while maintaining the crystalline nature of iron oxide nanoparticles.^33^ Similar structural characteristics have also been observed in GO/Fe_3_O_4_-containing hybrid nanocomposites developed for biomedical applications. The XRD patterns of GO/Fe_3_O_4_-modified clay composites demonstrated that the incorporation of magnetic nanoparticles did not disrupt the crystal structure of either graphene oxide or Fe_3_O_4_, confirming the successful formation of the hybrid material.^34^ Likewise, multifunctional GO@Fe_3_O_4_-based nanostructures retained the characteristic diffraction peaks of magnetite after further surface functionalization, indicating that the Fe_3_O_4_ crystalline phase remained stable throughout subsequent modification steps.^35^ The observed peak broadening is also consistent with previous studies on magnetic graphene-based nanocomposites, where broadened Fe_3_O_4_ reflections were attributed to the nanoscale crystallite size and the homogeneous dispersion of magnetic nanoparticles on graphene sheets.^37,38^ Similar XRD characteristics have additionally been reported for FeGO-based magnetic adsorbents and cellulose/graphene oxide magnetic composites, further confirming that the preservation of the magnetite diffraction peaks together with graphene-related reflections is a typical structural feature of successfully synthesized magnetic graphene oxide composites.^39,40^ Therefore, the excellent agreement between the present diffraction pattern and previous reports provides strong evidence for the successful synthesis of the magnetic graphene oxide composite and the effective integration of crystalline Fe_3_O_4_ nanoparticles within the graphene oxide matrix.

Compared with the magnetic activated carbon reference sample (Figure 3), the MGO composite exhibited the characteristic magnetite reflections together with graphene-related diffraction features, whereas the AC-based material primarily reflected the carbonaceous support. The similar Fe_3_O_4_ diffraction peaks observed in both magnetic materials confirm successful magnetite formation regardless of the support material, while the differences in the carbon-related reflections originate from the distinct structural organizations of graphene oxide and activated carbon. Unlike activated carbon, graphene oxide possesses a layered graphitic framework rich in oxygen-containing functional groups, which provide suitable anchoring sites for homogeneous Fe_3_O_4_ deposition and contribute to the formation of a more integrated hybrid structure.^30,33–35,39,40^

### 3.3 Textural Characterization (BET Analysis)

The N_2_ adsorption–desorption isotherm of the MGO composite is presented in Figure 4. The isotherm exhibits a typical Type IV classification with a distinct H3-type hysteresis loop according to the IUPAC nomenclature, which is a characteristic feature of mesoporous materials. The hysteresis loop observed in the relative pressure range of 0.4–1.0 indicates the presence of slit-like pores often associated with the layered structure of graphene oxide. According to BET analysis, the specific surface area of the MGO was determined to be 37.94 m^2^/g, with a high correlation coefficient (*r*) of 0.99996 and a *C* constant of 179.47. The total pore volume was calculated as 0.078 cm^3^/g for pores smaller than 82.27 nm (radius) at a relative pressure of 0.988. Furthermore, the average pore radius was found to be 4.12 nm, further confirming the mesoporous nature of the adsorbent. This mesoporous framework is particularly advantageous for the adsorption of organic acids, such as lactic, succinic, and citric acids, as it provides accessible active sites and facilitates the diffusion of these molecules into the internal pore structure. Textural properties of the MGO and AC are shown in Table 1.

**Figure 4:**
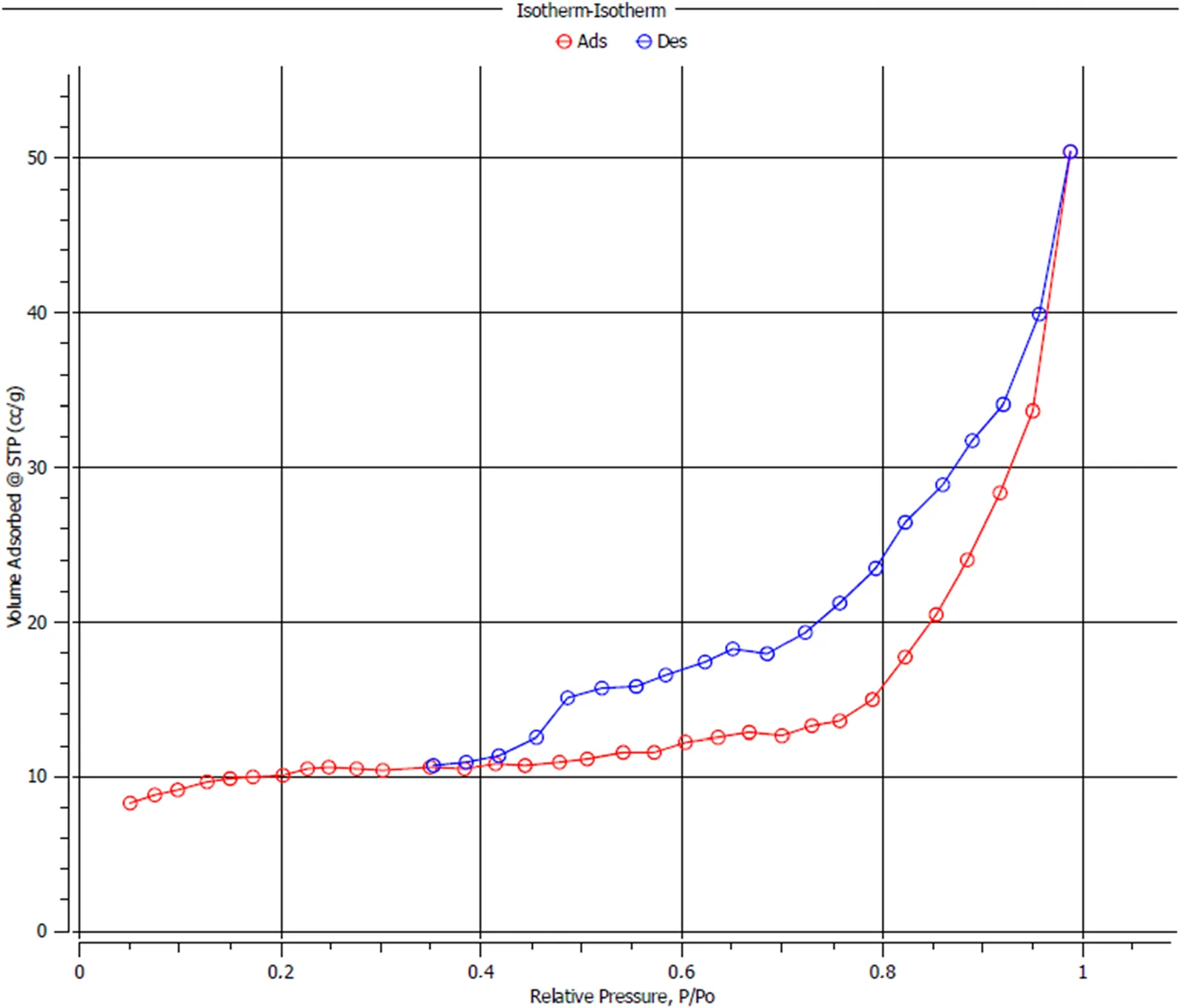
BET result of MGO.

**Table 1:** Textural Properties of the MGO and AC

	value	
property	MGO	AC
BET surface area (m^2^/g)	37.94	40.38
total pore volume (cm^3^/g)	0.078	0.1139
average pore radius (nm)	4.12	5.64
correlation coefficient (*r*)	0.99996	0.99993
*C* constant	179.47	98.88

The BET surface area of the synthesized MGO (37.94 m^2^/g) is relatively modest compared to some carbon-based materials; however, its exceptional adsorption capacity (*q*_max_ = 334.45 mg/g for lactic acid) confirms that the adsorption performance is governed by the synergistic effect of pore accessibility and surface chemistry rather than surface area alone. To provide a rigorous comparative basis, parallel textural characterization was conducted on a magnetic activated carbon (AC) reference, which exhibited a slightly higher surface area (40.38 m^2^/g), a larger total pore volume (0.1139 cm^3^/g), and a broader average pore radius (5.64 nm). Despite these theoretically superior textural parameters of the magnetic AC, its maximum adsorption capacity for lactic acid was substantially lower (*q*_max_ = 74.6 mg/g), which directly underscores that surface area is not the primary determinant of efficiency in these systems. The literature supports this observation; for instance, Jahan et al. reported a graphene oxide with a surface area of only 9.10 m^2^/g that achieved a massive methylene blue adsorption capacity of 3333 mg/g, proving that oxygen-containing functional groups provide the dominant driving force.^41^

In our study, while both materials possess mesoporous architectures, the superiority of MGO arises from its dense population of oxygen-rich moieties (hydroxyl, epoxy, and carboxyl groups), which facilitate specific hydrogen bonding and π–π interactions that are absent in the purely physical framework of the AC reference. This mesoporous architecture, comparable to other high-performance composites with pore diameters around 3.12–3.30 nm, ensures that high-concentration organic acid molecules can penetrate the internal framework and access these high-density functional groups without the “pore-clogging” issues often seen in microporous adsorbents.^42^ Consequently, MGO’s performance matches or exceeds specialized industrial resins like Amberlite IRA-67 (*q*_max_ = 333.33 mg/g), as summarized in Table 2. This comparison justifies the rationality of MGO’s design for large-scale biotechnological separations.^43^

**Table 2:** Comparison of Textural Parameters and Adsorption Capacities of MGO with the Literature

adsorbent	BET surface area (m^2^/g)	pore size/architecture	adsorbate	*q*_max_ (mg/g)	references
MGO	37.94	4.12 nm (mesopore)	lactic acid	334.45	this study
Amberlite IRA-67	not reported	gel-type resin	lactic acid	333.33	^43^
activated carbon		microporous	lactic acid	256.41	^43^
graphene oxide (GO)	9.10	mesoporous	methylene blue	3333	^41^
rGO composite	46.65	3.12 nm (mesopore)			^42^
magnetic AC	moderate	microporous	lactic acid	74.6	this study

Although the BET surface area of the synthesized MGO (37.94 m^2^/g) is relatively modest compared with some graphene-based adsorbents reported in the literature, the adsorption performance cannot be explained solely by the specific surface area. Instead, the adsorption efficiency is strongly influenced by the accessibility of adsorption sites, pore architecture, and surface chemistry. Type IV isotherm together with the average pore radius of 4.12 nm confirms the mesoporous nature of the material, which facilitates molecular diffusion and improves the accessibility of adsorption sites for organic acid molecules.^44,45^ Furthermore, graphene oxide contains abundant oxygen-containing functional groups capable of forming hydrogen bonding and other specific interactions with adsorbates, thereby contributing significantly to adsorption performance beyond the contribution of surface area alone.^44,46^

Previous studies have similarly demonstrated that increasing BET surface area does not necessarily result in enhanced adsorption capacity. For example, although isophthalic acid-modified HKUST-1 exhibited the highest BET surface area, excessive ultramicropore formation restricted molecular diffusion and resulted in lower methane uptake, highlighting that the pore size distribution and pore accessibility are often more critical than the surface area itself.^47^ Likewise, adsorption enhancement has been attributed to chemisorption, hydrogen bonding, electrostatic interactions, and π–π interactions rather than to surface area alone.^46,48,49^ Therefore, the moderate BET surface area observed in the present study is compensated by the favorable mesoporous structure together with abundant oxygen-containing functional groups, which collectively provide efficient adsorption sites for lactic, succinic, and citric acids. The present results suggest that adsorption capacity is governed by the synergistic effect of pore accessibility and surface chemistry rather than by BET surface area alone, which is consistent with previous studies on graphene-based adsorbents.^44,46,49^

### 3.4 Adsorption Isotherms of Organic Acids

#### 3.4.1 Succinic Acid

The effect of initial succinic acid concentration on adsorption capacity is summarized in Table 3, and Langmuir isotherm parameters are presented in Table 4. The corresponding isotherm plots are shown in Figure 5.

**Figure 5:**
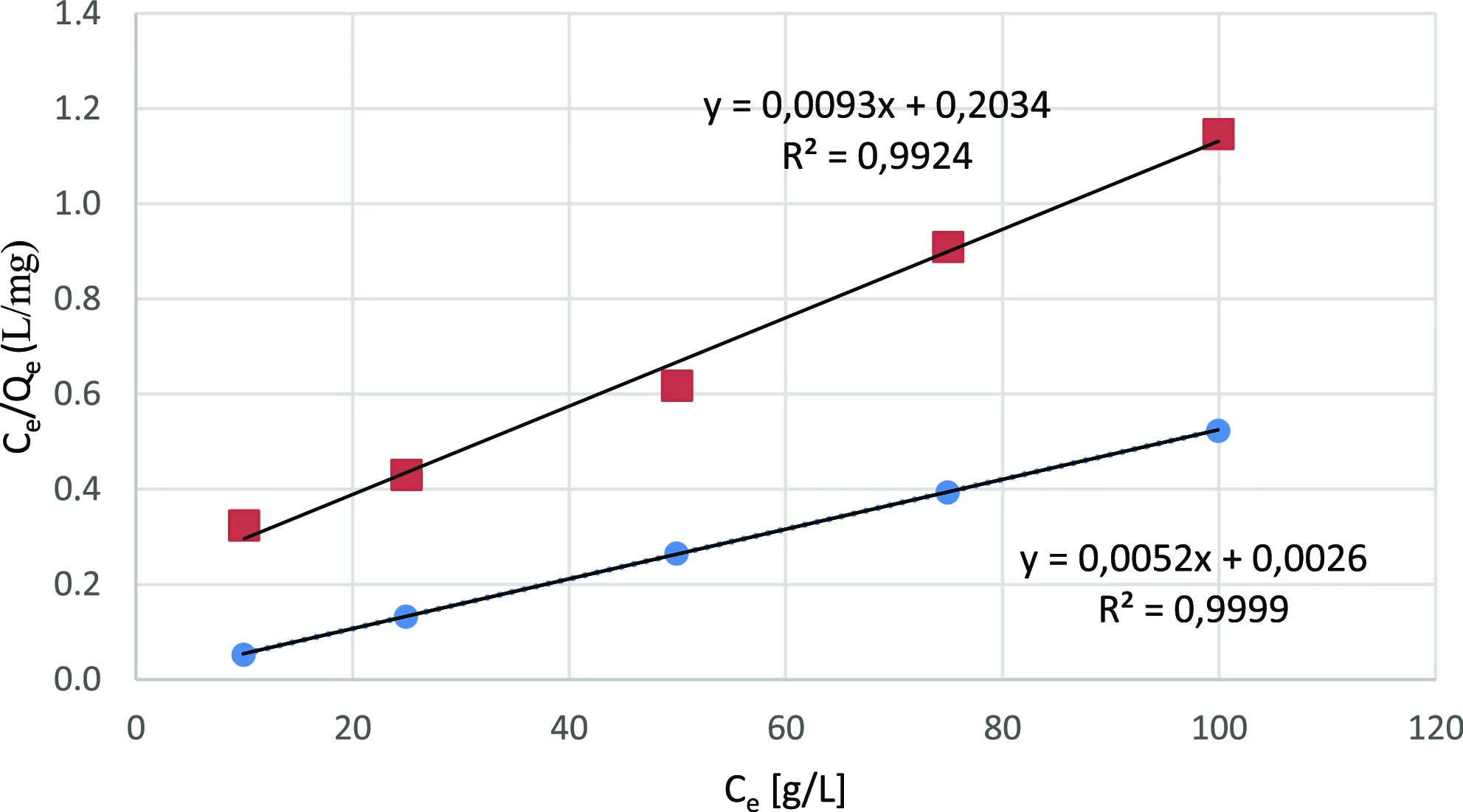
Isotherm model for succinic acid: red box solid, sample prepared with activated carbon; blue circle solid, sample prepared using graphene oxide.

**Table 3:** Effect of Initial Acid Concentration on Succinic Acid Adsorption

adsorbent	initial acid concentration, *C*_0_ (g/L)	equilibrium concentration, *C*_e_*^c^ (g/L)	adsorption capacity, *Q*_e_* (mg/g)
^a^AC-based	10	9.98 ± 0.001	30.8 ± 2.3
	25	24.97 ± 0.002	58.0 ± 3.2
	50	49.96 ± 0.002	80.8 ± 4.2
	75	74.96 ± 0.002	82.4 ± 3.9
	100	99.96 ± 0.002	87.2 ± 4.2
^b^MGO-based	10	9.91 ± 0.002	187.2 ± 4.2
	25	24.91 ± 0.002	187.2 ± 3.0
	50	49.91 ± 0.002	188.0 ± 4.7
	75	74.91 ± 0.003	190 ± 5.5
	100	99.90 ± 0.004	190.8 ± 8.7

a Denotes the equilibrium state values.

b AC-based: magnetic activated carbon-based.

c MGO-based: magnetically modified graphene oxide-based.

**Table 4:** Langmuir Isotherm Data of Succinic Acid

adsorbent	*R* ^2^	*q*_max_ (mg/g)	*K*_L_ (L/mg)	*R*_L_ (*C*_0_ = 1%)	*R*_L_ (*C*_0_ = 10%)
AC-based	0.99	107.53	0.0457	2.2 × 10^–3^	2.2 × 10^–4^
MGO-based	0.99	192.31	2.00	5 × 10^–5^	5 × 10^–6^

To quantitatively evaluate the succinic acid adsorption performance between MGO-based and AC-based adsorbents, a two-sample *t*-test was conducted on the data set presented in Table 3. The statistical analysis revealed highly significant differences between the two functional materials across the investigated concentration range (10–100 g/L). Specifically, the overall mean adsorption capacity (*Q*_e_) was determined as 188.6 mg/g for MGO-based and 67.8 mg/g for AC-based adsorbents, demonstrating a highly significant statistical variation (*p* < 0.001). Similarly, the analysis for equilibrium concentration (*C*_e_) yielded mean values of 51.9 g/L (MGO-based) and 52 g/L (AC-based), confirming the distinct mass balance profiles. These results clearly establish that the MGO composite provides a vastly superior affinity and saturation capacity for succinic acid recovery compared to the AC reference.

Both AC- and graphene oxide-based samples exhibit good agreement with the Langmuir isotherm model. The higher *q*_max_ value obtained for the MGO-based adsorbent indicates an enhanced adsorption capacity compared to AC.

#### 3.4.2 Citric Acid

Adsorption data for citric acid are presented in Table 5, and Langmuir isotherm parameters are summarized in Table 6. The isotherm plots are shown in Figure 6.

**Figure 6:**
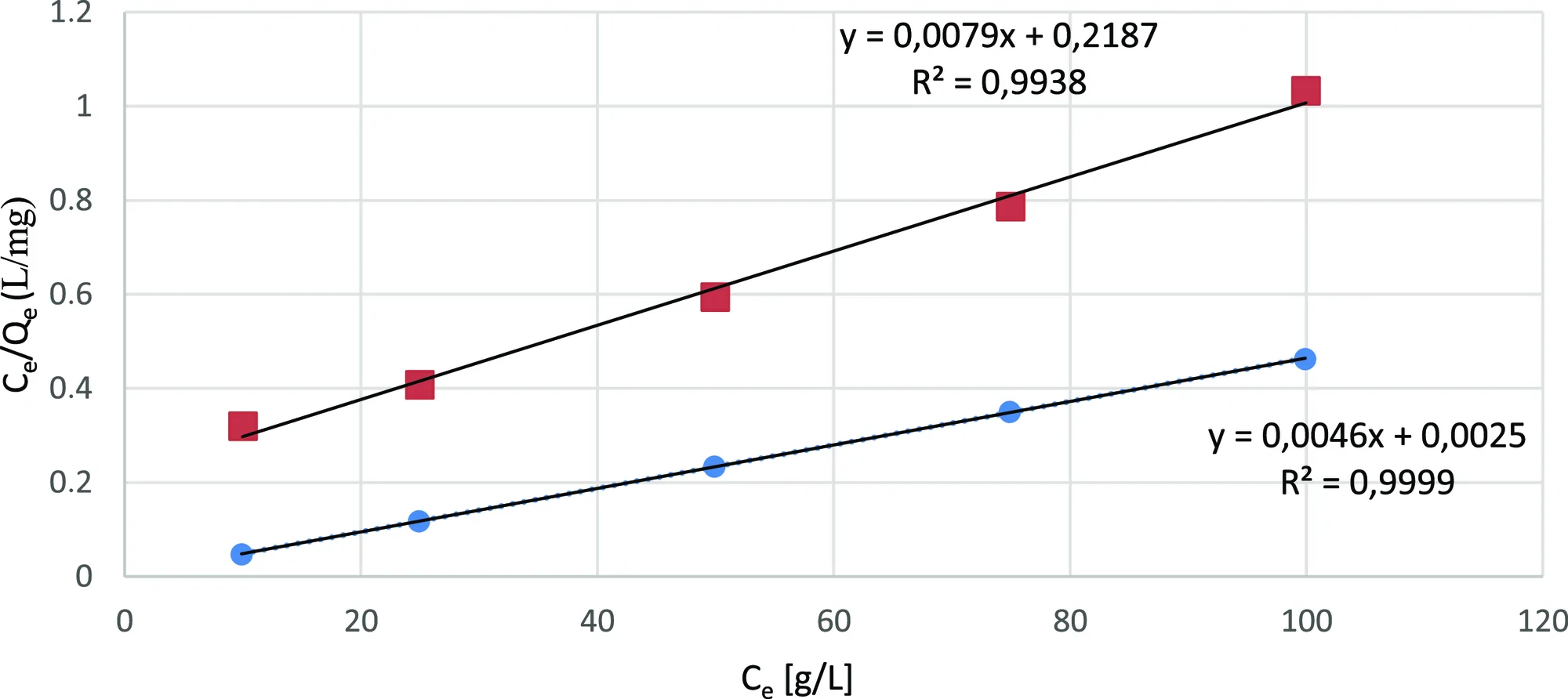
Isotherm model for citric acid: red box solid, sample prepared with activated carbon; blue circle solid, sample prepared using graphene oxide.

**Table 5:** Effect of Initial Acid Concentration on Citric Acid Adsorption

adsorbent	initial acid concentration, *C*_0_ (g/L)	equilibrium concentration, *C*_e_*^a^ (g/L)	adsorption capacity, *Q*_e_* (mg/g)
AC-based	10	9.98 ± 0.001	31.2 ± 2.3
	25	24.97 ± 0.001	61.2 ± 2.3
	50	49.96 ± 0.002	84.0 ± 3.2
	75	74.95 ± 0.001	95.2 ± 2.3
	100	99.95 ± 0.002	96.8 ± 3.0
MGO-based	10	9.89 ± 0.002	210.4 ± 3.9
	25	24.89 ± 0.001	211.6 ± 3.0
	50	49.89 ± 0.002	212.8 ± 3.4
	75	74.89 ± 0.002	214 ± 3.2
	100	99.89 ± 0.002	216 ± 3.7

a Denotes the equilibrium state values.

**Table 6:** Langmuir Isotherm Data of Citric Acid

adsorbent	*R* ^2^	*q*_max_ (mg/g)	*K*_L_ (L/mg)	*R*_L_ (*C*_0_ = 1%)	*R*_L_ (*C*_0_ = 10%)
AC-based	0.99	126.6	0.036	2.8 × 10^–3^	3 × 10^–4^
MGO-based	0.99	217.4	1.84	5 × 10^–5^	5 × 10^–6^

To quantitatively evaluate the citric acid adsorption performance between MGO-based and AC-based adsorbents, a two-sample *t*-test was conducted on the data set presented in Table 5. The statistical analysis revealed highly significant differences in adsorption capacities between the two materials across the investigated concentration range (10–100 g/L). Specifically, the overall mean adsorption capacity was determined to be 213 mg/g for MGO-based and 73.7 mg/g for AC-based adsorbents, showing high statistical significance (*p* < 0.001). The analysis for equilibrium concentration yielded mean values of 51.9 g/L (MGO-based) and 52 g/L (AC-based), confirming that while the mass balance remains consistent, MGO provides a much more efficient adsorption platform. This superiority is attributed to its mesoporous framework (4.12 nm), which effectively overcomes the microporous limitations of the AC reference.

The graphene oxide-based sample demonstrates a slightly higher adsorption capacity and better fitting to the Langmuir model compared with AC, suggesting improved interaction between citric acid molecules and the modified graphene surface.

#### 3.4.3 Lactic Acid

The adsorption behavior of lactic acid is summarized in Tables 7 and 8, with the Langmuir isotherm plot shown in Figure 7.

**Figure 7:**
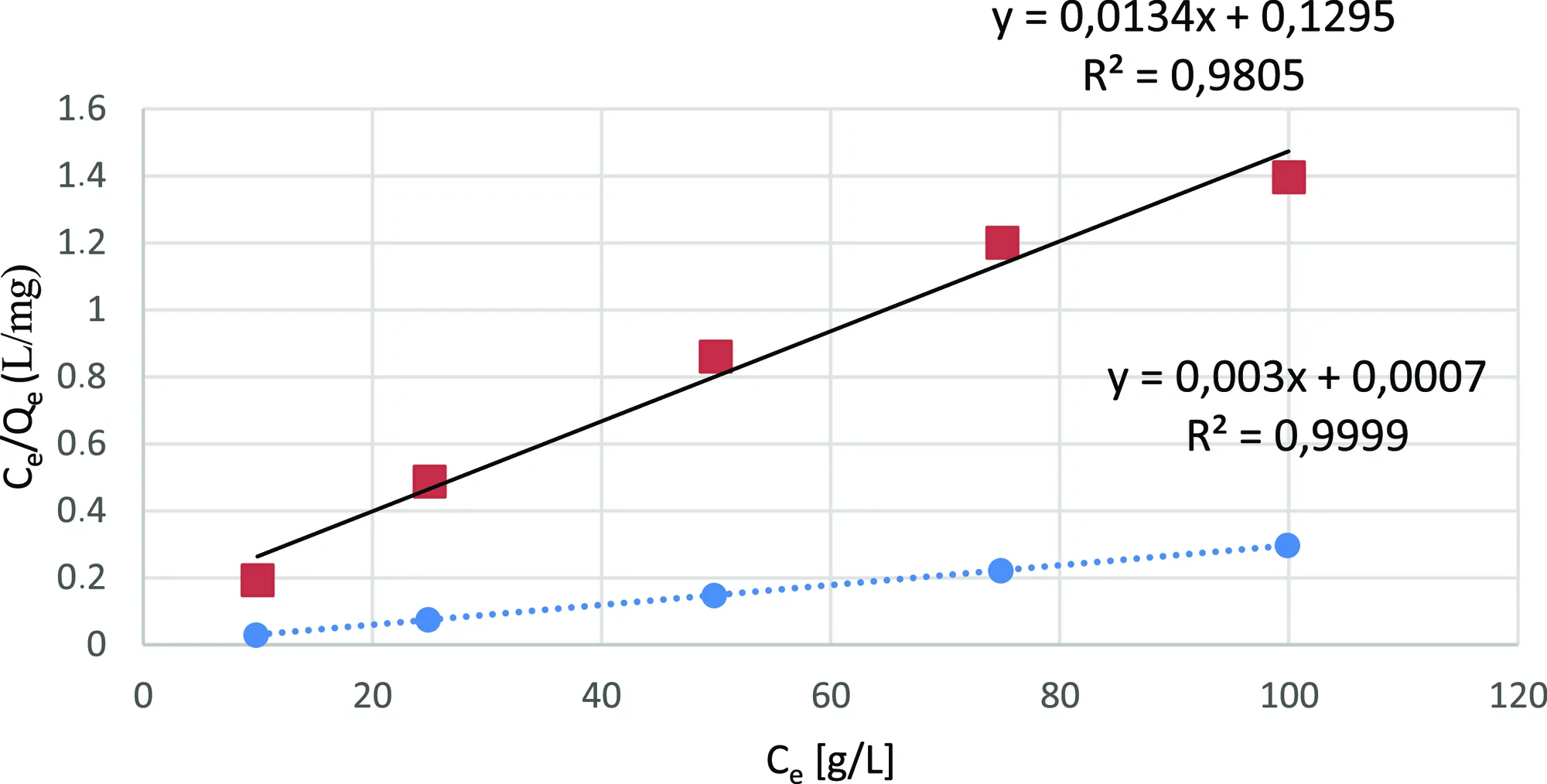
Isotherm model for lactic acid: red box solid, sample prepared with activated carbon; blue circle solid, sample prepared using graphene oxide.

**Table 7:** Langmuir Isotherm Data of Lactic Acid

adsorbent	*R* ^2^	*q*_max_ (mg/g)	*K*_L_ (L/mg)	*R*_L_ (*C*_0_ = 1%)	*R*_L_ (*C*_0_ = 10%)
AC-based	0.98	74.6	0.10	10^–3^	10^–4^
MGO-based	0.99	334.45	4.27	2.3 × 10^–5^	2.3 × 10^–6^

**Table 8:** Effect of Initial Acid Concentration on Lactic Acid Adsorption

adsorbent	initial acid concentration, *C*_0_ (g/L)	equilibrium concentration, *C*_e_ ^a^ (g/L)	adsorption capacity, *Q*_e_ ^a^ (mg/g)
AC-based	10	9.97 ± 0.004	51.6 ± 7.8
	25	24.97 ± 0.004	51.2 ± 7.3
	50	49.97 ± 0.003	58.0 ± 5.8
	75	74.97 ± 0.004	62.4 ± 7.8
	100	99.96 ± 0.003	71.6 ± 5.2
MGO-based	10	24.83 ± 0.002	326.8 ± 2.7
	25	24.83 ± 0.002	333.6 ± 5.0
	50	49.83 ± 0.003	337.2 ± 5.2
	75	74.83 ± 0.006	338.8 ± 12.1
	100	99.83 ± 0.002	336 ± 3.2

a Note: Statistical consistency was verified using a two-sample *t*-test for both adsorption capacity (*Q*_e_) and equilibrium concentration (*C*_e_). Across the investigated concentration range (10–100 g/L), statistically significant differences were observed between MGO-based and AC-based adsorbents (*p* < 0.001), particularly in the adsorption capacity.

It is noteworthy that at lower lactic acid concentrations, specifically below approximately 3 g/L, the activated carbon-based adsorbent exhibits a slightly higher adsorption capacity than the MGO composite (as seen in Table 8). However, as the equilibrium concentration increases beyond this point, the performance of the MGO framework becomes superior, eventually reaching a significantly higher theoretical maximum capacity (*q*_max_) compared with AC, as confirmed by the Langmuir model results. The higher *q*_max_ value suggests that the surface chemistry of graphene oxide provides favorable interaction sites for the adsorption of lactic acid.

The superior adsorption capacity of lactic acid (*q*_max_ = 334.45 mg/g) is mechanistically linked to the multifactorial coupling effect of its molecular structure and functional group density. Unlike the dicarboxylic (succinic) and tricarboxylic (citric) acids, lactic acid’s monocarboxylic nature results in a significantly smaller molecular footprint, which facilitates high-density packing and efficient pore-filling within the 4.12 nm mesoporous framework of the MGO. A critical quantitative driver for this dominance is the presence of the hydroxyl (−OH) group, which facilitates supplementary hydrogen bonding with the epoxy and carboxyl groups on the MGO surface, thereby providing a greater number of effective binding sites per unit area compared to the bulkier polycarboxylic counterparts. Furthermore, the relationship between the experimental pH (2.0–3.5) and the p*K*_a_ of lactic acid (3.86) ensures that the molecules exist predominantly in their neutral form, which minimizes electrostatic repulsion and allows short-range forces, specifically π–π stacking and hydroxyl-mediated hydrogen bonding to act as the dominant driving forces for achieving superior saturation limits. The absence of electrostatic repulsion at the working pH range (2.0–3.5) facilitates a higher packing density of neutral lactic acid molecules on the MGO sheets, as the surface’s oxygenated functional groups can engage in unrestricted multisite hydrogen bonding without being hampered by anionic charge interference.

To quantitatively evaluate the performance difference between MGO-based and AC-based adsorbents, a two-sample *t*-test was conducted on the data set presented in Table 8. The statistical analysis for adsorption capacity (*Q*_e_) showed mean values of 334.5 mg/g for MGO-based and 59 mg/g for AC-based adsorbents, revealing a highly significant difference (*p* < 0.001). This confirms the superior performance of the MGO composite across the entire concentration range. Similarly, the analysis for equilibrium concentration (*C*_e_) yielded mean values of 51.8 g/L (MGO-based) and 52 g/L (AC-based), reflecting the distinct mass balance profiles and pore-filling efficiencies of the two materials. This clear statistical significance is a direct consequence of the structural advantages of MGO; while AC-based adsorbents are limited by their microporous nature, the mesoporous framework of MGO (pore radius: 4.12 nm) facilitates high-density functional group accessibility and higher saturation limits. Consequently, the statistical results confirm MGO’s superior *q*_max_ capability (334.45 mg/g) for concentrated acid recovery compared to traditional carbon-based references.

To further elucidate the adsorption mechanism and surface heterogeneity, we fitted the experimental data to the Freundlich isotherm model. As presented in Table 9, the Freundlich constant (*K*_F_), which relates to the adsorption capacity, was generally higher for the MGO composite compared to the AC reference for succinic acid, confirming the enhanced performance of the graphene-based adsorbent. The heterogeneity factor (1/*n*) provides crucial information regarding the adsorption intensity and the surface energy distribution. For all of the investigated organic acids, the 1/*n* values were found to be between 0 and 1, ranging from 0.01 to 0.50. According to the Freundlich theory, 1/*n* values in this range indicate a favorable adsorption process and suggest that the MGO surface possesses a distribution of active sites with varying affinities. Specifically, for lactic acid on MGO, the high correlation coefficient (*R* ^2^ = 0.826) and the 1/*n* value of 0.01 suggest that while the surface is relatively homogeneous (as also indicated by the excellent Langmuir fit), the functional group density on the graphene oxide sheets facilitates multilayer interactions or cooperative binding. This supports the observation of the highest maximum capacity (*q*_max_ = 334.45) for lactic acid, likely driven by its ability to utilize a broader range of oxygenated sites through its hydroxyl and carboxyl groups. The successful fitting of both the Langmuir and Freundlich models confirms that the MGO composite serves as a robust platform for the recovery of these biotechnologically relevant acids.

**Table 9:** Freundlich Isotherm Parameters for the Adsorption of Organic Acids onto AC and MGO

organic acid	adsorbent	*K*_F_ [(mg/g) (L/mg)^1/*n*^]	1/*n*	*R* ^2^
succinic acid	AC-based	12.04	0.45	0.930
	MGO-based	183	0.01	0.759
citric acid	AC-based	10.91	0.50	0.950
	MGO-based	205	0.01	0.899
lactic acid	AC-based	35.7	0.13	0.787
	MGO-based	318	0.01	0.826

The maximum adsorption capacity of MGO for lactic acid (334.45 mg/g) is highly competitive and comparable to that of specialized industrial adsorbents. Specifically, previous research reported a nearly identical *q*_max_ value of 333.33 mg/g for the weakly basic ion-exchange resin Amberlite IRA-67 during lactic acid recovery from model fermentation broths.^43^ This similarity underscores the efficiency of the synthesized MGO composite as a high-performance alternative to organic acid separation.

To evaluate the interaction characteristics between the organic acids and the MGO surface, the experimental data were further analyzed using the Temkin isotherm model. As shown in Table 10, the Temkin model provided a satisfactory fit for all investigated organic acids, with correlation coefficients (*R* ^2^) ranging from 0.758 to 0.897 for the MGO-based adsorbent. The Temkin constant *A*, representing the equilibrium binding parameter, was highest for succinic acid on MGO (8.79 × 10^47^), suggesting comparatively stronger initial interactions between dicarboxylic species and the modified adsorbent surface. The Temkin constant *B*, associated with adsorption heat distribution, was consistently higher for AC-based reference than for MGO across all acids, indicating a broader distribution of adsorption energies on the AC-based reference. The adsorption mechanism on the MGO platform arises from a synergistic interplay of several distinct forces. Pore-filling within the 4.12 nm mesoporous structure provides a physical basis for the recovery, while π–π interactions between the functional groups (carbonyl/hydroxyl) of the organic acids and the graphitic framework significantly strengthen the binding affinity. Furthermore, electrostatic attraction occurring between partially dissociated carboxylate groups and the surface, complemented by extensive hydrogen bonding with oxygen-rich functional groups, plays a critical role in the process. The high correlation with the Temkin model (*R* ^2^ = 0.826 for lactic acid) further confirms that these heterogeneous surface interactions are the primary drivers governing the adsorption behavior. Similar applicability of the Temkin model for graphene oxide-containing adsorbents has been reported previously, emphasizing the role of heterogeneous surface interactions in adsorption systems.^50^ In particular, the relatively high *B* value observed for citric acid on AC (29.69) may be associated with the presence of multiple carboxylic groups, enabling stronger multisite interactions with oxygen-containing surface functionalities. Furthermore, the satisfactory fit of the Temkin model for lactic acid suggests that the adsorption process is influenced by adsorbate surface interactions that vary with surface coverage. The enhanced adsorption behavior of MGO relative to AC may be associated with oxygen-containing functional groups and iron oxide incorporation, which can facilitate hydrogen bonding, electrostatic interactions, and surface complexation with carboxylic acid molecules. Given the solution pH and the p*K*_a_ values of the acids, the presence of both neutral and partially dissociated species allows for a complex interplay of forces. Specifically, at pH values below the p*K*_a_, the dominance of the neutral state transforms the MGO surface from a potential electrostatic repellent into a highly receptive platform for specific chemical interactions, which is the theoretical foundation for the high recovery rates observed across all investigated acids. Beyond hydrogen bonding, electrostatic interactions and surface complexation with the MGO surface play a significant role in this environment. Similar observations have been reported for graphene oxide-based adsorption systems, where surface chemistry was shown to play a more significant role than the surface area in governing the adsorption behavior.^31,51^ Additionally, the planar graphene oxide framework may provide improved surface accessibility compared with AC. Comparable strong adsorbate–surface interactions and rapid adsorption behavior have also been reported for graphene oxide-derived materials.^51–53^

**Table 10:** Temkin Isotherm Parameters for the Adsorption of Organic Acids onto AC and MGO

organic acid	adsorbent	*A* (L/g)	*B*	*R* ^2^
succinic acid	AC-based	3.8 × 10^–4^	25.02	0.966
	MGO-based	8.79 × 10^47^	1.56	0.758
citric acid	AC-based	3 × 10^–4^	29.69	0.987
	MGO-based	2.11 × 10^37^	2.21	0.897
lactic acid	AC-based	0.04	7.98	0.763
	MGO-based	1.34 × 10^27^	4.58	0.826

#### 3.4.4 Adsorption Kinetics

The kinetic parameters derived from the pseudo-second-order model are summarized in Table 11.

**Table 11:** Kinetic Parameters for the Adsorption of Organic Acids onto MGO^a^

organic acid	*q*_e,exp_ (mg/g)	*q*_e,cal_ (mg/g)	*k*_2_ (g/mg·min)	*R* ^2^ (PSO)
lactic acid	337.2	357.14	0.007	0.9964
succinic acid	188	196.08	0.0023	0.9967
citric acid	212.8	217.39	0.0019	0.9974

a *q*_e,exp_ and *q*_e,cal_ represent the experimental and calculated equilibrium adsorption capacities, respectively. *k*_2_ is the pseudo-second-order rate constant. All experiments were conducted at an initial concentration (*C*_0_) of 50 g/L, temperature of 25 °C, and an adsorbent dosage of 10 mg.

The adsorption kinetics and mechanism modeling of lactic, succinic, and citric acids on MGO composites are detailed in Figure 8.

**Figure 8:**
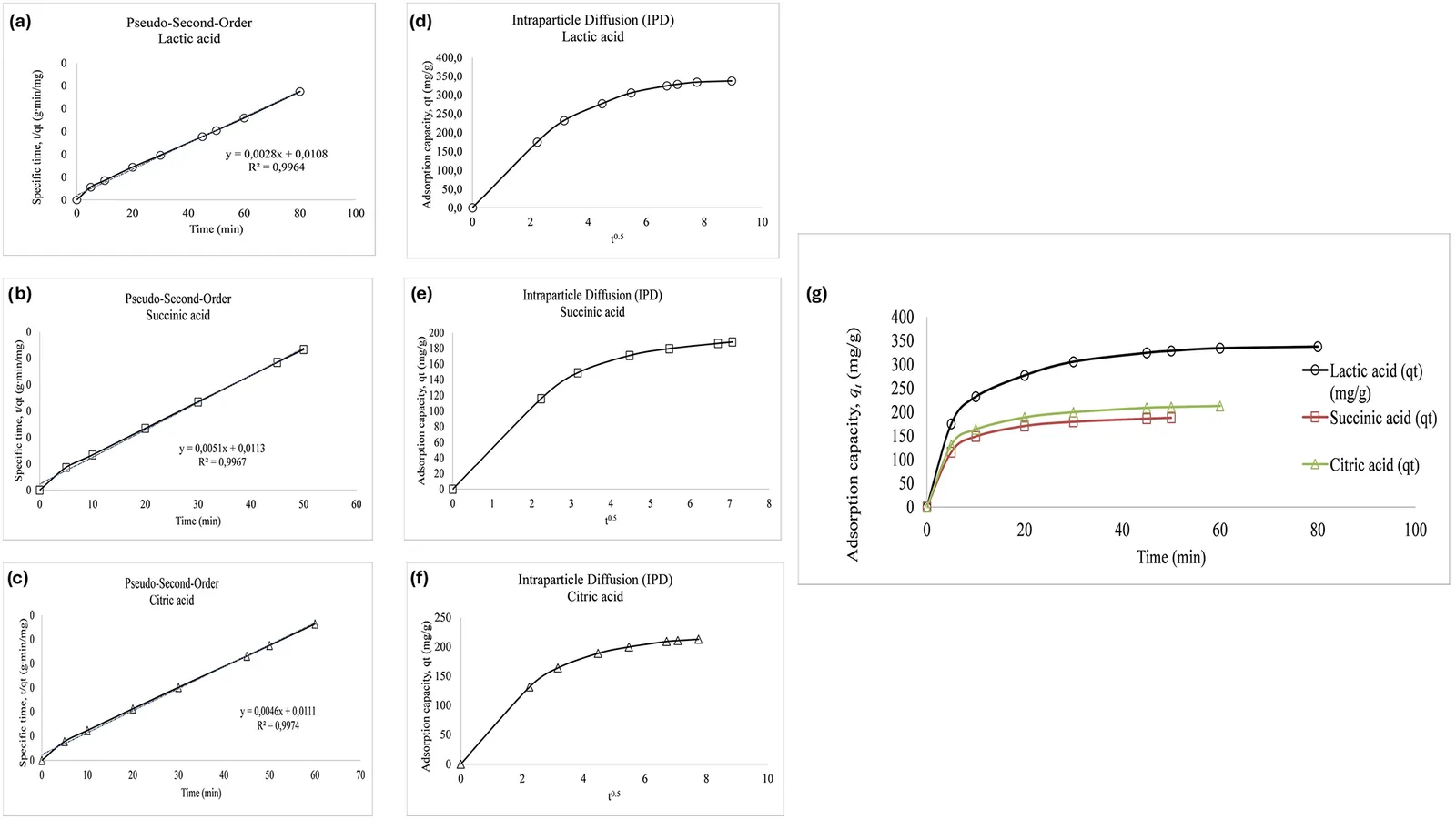
Adsorption kinetics and mechanism modeling of organic acids onto MGO. Adsorption kinetics of organic acids: (a–c) pseudo-second-order (PSO) kinetic model plots for lactic, succinic, and citric acids; (d–f) intraparticle diffusion (IPD) model plots for lactic, succinic, and citric acids; and (g) combined time-dependent adsorption capacity (*q*_*t*_) profile for the investigated acids.

The experimental data for all acids exhibited an excellent fit with the PSO model (*R* ^2^ > 0.99), as shown in Table 11 and Figure 8a–c. This confirms that the adsorption rate is governed by chemical interactions (chemisorption). The rate constants (*k*_2_) were determined as 0.007, 0.0023, and 0.0019 g/mg·min for lactic, succinic, and citric acids, respectively.

The lowest *k*_2_ value observed for citric acid (0.0019 g/mg·min) is quantitatively consistent with its bulky tricarboxylic structure, which causes significant steric hindrance during diffusion into the 4.12 nm mesoporous framework of the MGO. A comparative analysis of the rate constants decreasing from lactic (0.007 g/mg·min) and succinic (0.0023 g/mg·min) to citric acid (0.0019 g/mg·min) demonstrates a clear inverse correlation between molecular complexity and kinetic performance. Specifically, the increase in the number of carboxyl groups and the resulting increase in molecular volume elevate the steric resistance encountered during the penetration of the internal pore structure, thereby reducing the overall adsorption rate. This quantitative trend confirms that within the mesoporous MGO system, the multifactorial coupling of molecular footprint and functional group density acts as a primary constraint on the kinetics of mass transfer, where simpler molecular structures exhibit faster surface arrival and site-binding dynamics. Conversely, while lactic acid is the smallest molecule, it requires the longest equilibrium time (80 min) because it achieves the highest total loading (*q*_max_ = 334.45 mg/g), requiring more time to saturate the oxygen-rich active sites. Furthermore, the IPD plots (Figure 8d–f) confirmed a multistage diffusion process within the 4.12 nm mesopores of MGO.

To further elucidate the diffusion mechanism, the boundary layer constants (*C*) and intraparticle diffusion rate constants (*k*_id_) were quantitatively evaluated (Table 12).

**Table 12:** Intraparticle Diffusion Model Constants for Organic Acids onto MGO

organic acid	molecular structure	*k*_id_ (mg/g·min^0^·^5^)	*C* (mg/g)	*R* ^2^
lactic acid	monocarboxylic	39.26	96.76	0.971
succinic acid	dicarboxylic	19.14	80.33	0.928
citric acid	tricarboxylic	20.80	91.01	0.948

The nonzero intercepts (*C*) obtained for all acids confirm that the adsorption process is not solely governed by intraparticle diffusion but involves a combination of film diffusion and internal pore diffusion. The highest *C* value observed for lactic acid (96.76 mg/g) suggests that its smaller molecular footprint and additional hydroxyl group facilitate rapid initial accumulation at the boundary layer. Conversely, the *k*_id_ values decrease significantly as the number of carboxyl groups increases (lactic > citric ≈ succinic), illustrating that steric hindrance and hydrated molecular volume for tricarboxylic species like citric acid act as critical constraints during the penetration into the internal 4.12 nm mesopores of the MGO.

To further assess adsorption favorability, the dimensionless separation factor (*R*_L_) was calculated for each acid–adsorbent system. The *R*_L_ parameter, derived from the Langmuir model, indicates whether adsorption is unfavorable (*R*_L_ > 1), linear (*R*_L_ = 1), favorable (0 < *R*_L_ < 1), or irreversible (*R*_L_ = 0). As summarized in Tables 4, 6, and 7, *R*_L_ values for all investigated acids on both adsorbents across the studied concentration range (10–100 g/L) were between 2.3 × 10^–6^ and 2.8 × 10^–3^.

Since all *R*_L_ values remained within the favorable range (0 < *R*_L_ < 1), the adsorption of lactic, succinic, and citric acids onto both synthesized adsorbents can be considered favorable under the investigated conditions. The observed decrease in *R*_L_ with an increasing initial concentration suggests progressively more favorable adsorption at higher loading concentrations, indicating the applicability of these materials under concentrated process conditions.

The comparatively high Langmuir capacity parameter observed for lactic acid may be associated with its smaller molecular size and the presence of an additional hydroxyl group, which may facilitate supplementary hydrogen bonding interactions with the oxygen-functionalized graphene oxide surface. A similar enhancement of adsorption affinity through surface functionalization has been reported for modified graphene oxide systems.^31,54^

Comparative evaluation of the three investigated acids indicates that the adsorption behavior is influenced by the molecular structure and functional group composition. The MGO-based adsorbent consistently exhibited an adsorption performance higher than that of the AC-based material, particularly for lactic acid. Differences in adsorption behavior may arise from variations in the carboxyl group number, molecular size, and interaction mechanisms such as hydrogen bonding and electrostatic interactions.

### 3.5 Stability and Reusability of MGO

The reusability of an adsorbent is a critical index for its industrial viability and sustainability in large-scale downstream separation processes. Experimental results for five consecutive cycles demonstrated that the synthesized MGO maintains excellent structural stability and performance. The adsorption capacity for lactic acid exhibited a high retention rate, decreasing only slightly from its initial value to 91.4% by the end of the fifth cycle. This minor loss is attributed to the unavoidable occupation of some high-energy active sites or minor mass loss during the magnetic separation and washing steps, a phenomenon frequently reported for graphene-based magnetic composites.^55^ To ensure a comprehensive evaluation of MGO’s industrial robustness, parallel five-cycle stability tests were performed for succinic and citric acids. The capacity retention rates after the fifth cycle were found to be 87.8% for succinic acid and 84.2% for citric acid. The relatively higher capacity loss observed for these polycarboxylic species compared to lactic acid (91.4%) is mechanistically attributed to multicarboxylate-induced active site fouling. Due to the presence of multiple carboxyl groups, succinic and citric acids can facilitate multidentate attachment through simultaneous hydrogen bonding and electrostatic interactions with the hydroxyl and epoxy moieties of the MGO framework. This multisite coordination creates a more persistent surface bond that is partially resistant to the NaOH-mediated regeneration process, resulting in the cumulative occupation of some active binding sites. Nevertheless, the high overall stability (>84%) confirms that the MGO composite maintains its structural integrity and remains a sustainable alternative for the large-scale separation of complex platform chemicals. This retention performance is highly competitive when compared to other advanced adsorbents reported in recent studies. For instance, Wang et al. reported that an rGO-based catalytic membrane maintained removal efficiencies above 90% across five filtration cycles.^56^ Additionally, Mustafa et al. demonstrated that GO-reinforced composites can exhibit up to 95% reusability after multiple cycles, justifying the long-term stability of such frameworks.^57^ The observed low capacity loss (averaging 1.7% per cycle) is quantitatively consistent with the findings of Xiong et al., who reported that biochar-graphene oxide composite monoliths remained highly stable over five cycles with an average loss of only 2.7% per cycle.^58^

The magnetic nature of the composite facilitated rapid recovery from the aqueous phase within less than 30 s. This consistent and rapid magnetic performance serves as a critical functional validation of the successful structural integration of magnetite nanoparticles within the graphene oxide framework. The fact that the material maintains its swift magnetic response throughout multiple reusability cycles confirms that the magnetic phase is firmly and stably anchored to the GO sheets. This provides practical evidence of the composite’s integrity and synthesis success, effectively complementing the structural findings from XRD and FTIR even in the absence of direct microscopic visualization. This visual magnetic response supports efficient phase separation without the need for additional filtration or centrifugation steps, which are often considered challenging recovery methods in the literature.^57,59,60^ The synergy between the GO sheets and Fe_3_O_4_ nanoparticles not only provides this magnetic advantage but also enhances the mechanical robustness of the entire framework, as confirmed by Babakhani et al., where the presence of GO trapping within the matrix increased mechanical stability through interlocking structures.^34^ This rapid separation and high-performance retention confirm that MGO is a sustainable and cost-effective alternative for the separation of biotechnological organic acids, effectively addressing the research gap noted in earlier studies where regeneration was not explicitly investigated.^43^

## 4 Conclusion

In this study, an MGO composite was successfully synthesized and evaluated for the efficient recovery of platform organic acids. The integration of BET textural analysis alongside XRD and FTIR provided a comprehensive characterization, confirming the mesoporous nature of the adsorbent with a specific surface area of 37.94 m^2^/g, a total pore volume of 0.078 cm^3^/g, and an average pore radius of 4.12 nm. These properties, combined with the successful anchoring of Fe_3_O_4_ nanoparticles, facilitated enhanced molecular diffusion and provided robust magnetic separability from aqueous phases. Adsorption equilibrium data were systematically analyzed using Langmuir, Freundlich, and Temkin models to ensure a deeper understanding of the surface interactions. While the Langmuir model demonstrated excellent agreement (*R* ^2^ > 0.95), suggesting predominantly monolayer adsorption on a homogeneous distribution of active sites, the calculated dimensionless separation factor (*R*_L_) values (ranging from 2.3 × 10^–6^ to 2.8 × 10^–3^) confirmed that the adsorption process is highly favorable for all investigated acids. Furthermore, the successful fitting of the Freundlich and Temkin models indicated a diverse energy distribution of oxygenated functional groups on the MGO surface, which contributed to the high adsorption capacities compared to the AC reference. The findings clarify that the recovery performance is governed by functional group density rather than surface area alone. The hydroxyl-assisted hydrogen bonding in lactic acid provides a distinct advantage over the di- and tricarboxylic structures of succinic and citric acids. Among the tested adsorbates, lactic acid exhibited the highest maximum adsorption capacity (*q*_max_ = 334.45), which is mechanically attributed to its smaller molecular footprint and the specific interaction of its hydroxyl (−OH) group with the graphene oxide framework through hydrogen bonding. The overall findings underscore the potential of MGO as a sustainable and high-performance adsorbent for the biotechnology industry, offering rapid magnetic recovery and high efficiency for organic acid separation. Furthermore, the industrial viability of the MGO composite was confirmed through cyclic regeneration studies, which demonstrated that the material maintains a high capacity retention rate of 91.4% after five consecutive cycles. The rapid magnetic recovery (within <30 s) and the stability of its oxygen-rich framework ensure that MGO is a sustainable and cost-effective alternative for the large-scale separation of biotechnological platform chemicals.
